# Forensic use of Y-chromosome DNA: a general overview

**DOI:** 10.1007/s00439-017-1776-9

**Published:** 2017-03-17

**Authors:** Manfred Kayser

**Affiliations:** Department of Genetic Identification, Erasmus MC University Medical Center Rotterdam, PO Box 2040, 3000 CA Rotterdam, The Netherlands

## Abstract

The male-specific part of the human Y chromosome is widely used in forensic DNA analysis, particularly in cases where standard autosomal DNA profiling is not informative. A Y-chromosomal gene fragment is applied for inferring the biological sex of a crime scene trace donor. Haplotypes composed of Y-chromosomal short tandem repeat polymorphisms (Y-STRs) are used to characterise paternal lineages of unknown male trace donors, especially suitable when males and females have contributed to the same trace, such as in sexual assault cases. Y-STR haplotyping applied in crime scene investigation can (i) exclude male suspects from involvement in crime, (ii) identify the paternal lineage of male perpetrators, (iii) highlight multiple male contributors to a trace, and (iv) provide investigative leads for finding unknown male perpetrators. Y-STR haplotype analysis is employed in paternity disputes of male offspring and other types of paternal kinship testing, including historical cases, as well as in special cases of missing person and disaster victim identification involving men. Y-chromosome polymorphisms are applied for inferring the paternal bio-geographic ancestry of unknown trace donors or missing persons, in cases where autosomal DNA profiling is uninformative. In this overview, all different forensic applications of Y-chromosome DNA are described. To illustrate the necessity of forensic Y-chromosome analysis, the investigation of a prominent murder case is described, which initiated two changes in national forensic DNA legislation both covering Y-chromosome use, and was finally solved via an innovative Y-STR dragnet involving thousands of volunteers after 14 years. Finally, expectations for the future of forensic Y-chromosome DNA analysis are discussed.

## Making the case with a case

May 30, 1999, Queensday in The Netherlands: As every year, the Dutch were celebrating their Queen’s birthday with concerts, flea markets, and public and private parties all over the country. Marianne Vaatstra, a 16-year-old girl from the small village Zwaagwesteinde in the province Friesland, went for partying to the nearby village Kollum, from where she never returned home alive. On her bicycle ride back home at night, she was raped and murdered nearby the village Veenklooster, with her throat being slit, and traces of semen found in and on her body. No human eyewitness was available. No hit of the standard autosomal DNA profile obtained from the semen stains was found in the national criminal offender DNA database, which started in 1997 and, therefore, only included a few hundred persons by mid-1999.

A suspect from Zwaagwesteinde was arrested weeks later, but got released soon after, because his standard autosomal DNA profile did not match the one from the semen trace. Due to the murder scene’s location in close proximity to a political asylum seeker centER, the investigation also focused on the asylum seekers from this center. A man from Iraq, who left the center in the night of the murder and, therefore, raised suspicion, was tracked down by INTERPOL in Istanbul, but found innocent because of his non-matching standard autosomal DNA profile; as was a man from Afghanistan. By December 1999, approximately 150 persons, whom the investigators somehow linked with the case (but without enough evidence to make them case suspects), were voluntarily asked for a DNA sample; none of their standard autosomal DNA profiles matched the one obtained from the semen trace.

Soon after the crime happened, the local population strongly expressed its belief that the perpetrator must be one of the asylum seekers from the center, if only because of the assumed to be non-European manner of slit-throat murder. This led to serious conflicts between the local population and asylum seekers in the center as well as between the local population and police authorities. In the difficult situation of increasing social unrest, the public prosecutor in charge of the case turned to Peter de Knijff from the Forensic Laboratory for DNA Research (FLDO), Department of Human Genetics, Leiden University Medical Center. Ordered by the public prosecutor, FLDO obtained a Y-chromosome STR profile from the semen trace to infer the trace donor’s paternal bio-geographic ancestry. By comparing it with those stored in the Y-chromosome Haplotype Reference Database (YHRD) (www.yhrd.org), as well as others (published and unpublished) available to him, de Knijff concluded that the semen donor’s paternal ancestors are likely to originate from North-western Europe. With this result, it became clear to the investigators that they should rather widen their search for the unknown perpetrator among the Dutch European population. Although many local people were still reluctant to believe that the murderer was one of them, the results of this Y-chromosome bio-geographic ancestry test calmed down some of the social unrest in the region. However, the perpetrator was not found, and the Vaatstra case became a cold case for many years, until a different forensic use human Y-chromosome DNA eventually allowed for solving this murder case, albeit not before 14 years after it occurred.

This forensic use of Y chromosome DNA was remarkable in two ways. First, although, at that time, Y-STR profiling for paternal lineage identification was already introduced to forensics, for which Peter de Knijff together with Lutz Roewer from the Institute of Legal Medicine, Charité University Medicine Berlin were the leading scientists (de Knijff et al. 1997; Kayser et al. 1997, Roewer et al. 1992, 1996; Roewer and Epplen 1992), its forensic use for bio-geographic ancestry inference was not. Second, at that time in The Netherlands, forensic DNA analysis was regulated by a law from 1994, under which only autosomal STR profiling was legally allowed, but DNA inference of bio-geographic ancestry was not.

The investigation of the Vaatstra case is unique in the way that it stimulated two national law adaptations, both covering the forensic use of Y-chromosome DNA, albeit for different purposes. In 2003, likely stimulated by the Vaatstra case and the previous attempts to solve it including the illegally applied Y-chromosome ancestry testing, the Dutch parliament approved the first adaptation of the forensic DNA legislation. This law allows and regulates the forensic use of DNA information regarding bio-geographic ancestry and externally visible characteristics for investigative intelligence purposes to find unknown perpetrators of crime that cannot be identified by any other means. Moreover, in April 2012, the Dutch DNA legislation was adapted for a second time, allowing the forensic use of DNA for familial searching. Familial searching typically refers to the use of DNA evidence to find in criminal offender or suspect databases relatives of unknown perpetrators, whose standard autosomal DNA profiles are not yet included in such database and who, therefore, cannot be identified with DNA directly.

Two active ways of DNA-based familial searching regulated by this law were generally suitable to the Vaatstra case, and were thus applied to the case soon after the law adaptation was put in place. The first is searching the standard autosomal STR profiles of known offenders stored in the national DNA database for those that show strong similarity to the one from the crime scene trace. This approach can highlight close relatives of an unknown perpetrator being already included in the DNA database, which provides that investigative leads to eventually find the unknown perpetrator not yet included in the DNA database. Because of the use of autosomal STRs in standard DNA profiling, this approach is most suitable to trace close relatives (parents, children, and siblings). Distant relatives are difficult, if not impossible, to be traced with autosomal STRs due to occurring DNA recombination events that produce dissimilarities with every subsequent generation. Applying this approach in the Vaatstra case in 2012, when 142,120 persons were included in the national offender DNA database, revealed 121 men for whom an increased probability to be related with the unknown perpetrator was estimated.

Next, the special force team from police and prosecution, including forensic coordinator Ron Rintjema and tactical coordinator Jelle Tjalsma, in collaboration with Charissa van Kooten and Arnoud Kal from the Netherlands Forensic Institute (NFI) performed Y-STR profiling in selected men included in the national DNA database. In The Netherlands, DNA samples of persons from the national DNA database are kept, instead of being destroyed after standard DNA profiling as in some other countries. This is because the investigative use of the DNA samples from persons whose STR profile is stored in the national DNA database, involving additional DNA testing, is legally allowed. For the purpose of familial searching in the Vaatstra case, DNA samples from males included in the DNA database were selected for Y-STR analysis based on the following criteria: (i) they were previously identified as potential relatives via familial searching with autosomal STR profiling (121 males), (ii) they were born in or resident of the area in which the crime was committed (421 males), (iii) they carry region-specific surnames—present in the area where the crime was committed, but rare in The Netherlands as a whole (260 males). The latter criterion was applied because in patrilineal societies, as all European populations are, surnames and Y-chromosomes follow the same paternal mode of inheritance (see below). However, Y-STR profiling of these 802 criminal offenders did not reveal any complete or close match with the semen trace. This finding led to the conclusion that no close or distant paternal male relative of the unknown murderer of Marianne Vaatstra was included within that group of selected persons from the DNA database.

The second way of legally allowed familial searching is large-scale, voluntary DNA mass screenings (also called DNA dragnets) in the restricted geographic region where the crime occurred, assuming that the perpetrator does not participate. This is only allowed under certain circumstances such as serious crime leading to many years of imprisonment, and is particularly meant as last resort to solve cold cases where all other attempts have already failed (including the first described approach of familial search). From the tactical police investigation in the Vaatstra case, it was concluded that the perpetrator likely comes from the region. In September 2012, a large-scale DNA dragnet was decided as last resort to solve the Vaatstra case. More than 7600 men who lived in the region 5 km around the murderer site were invited to voluntarily provide a cheek swab sample for DNA analysis, and more than 6600 local men (87%) participated. Importantly, instead of using standard autosomal STR profiling, the special force team together with the NFI decided to apply Y-STR profiling in this DNA dragnet. Under the assumption that the unknown male perpetrator himself will not participate, it makes perfect sense from a scientific and policing perspective to carry out Y-STR profiling to find a male relative of the perpetrator, who, in turn, can guide the investigation to find the non-participating perpetrator. This is because Y-STR profiling in principle allows for highlighting all participating paternal male relatives of an unknown male perpetrator, close and distant ones, who typically share the same Y-STR profile, whereas autosomal STR profiling can only trace close relatives. The regional population and, thus, all volunteers participating in the dragnet were well informed by the authorities on the content and consequences of such Y-STR-based kinship approach via distributed brochures, leaflets, and a dedicated Website.

Y-STR profiling at 17 Y-STR markers using the commercial AmpFlSTR^®^ Yfiler^®^ kit (Thermo Fisher Scientific) (Table 1) was applied. Remarkably, however, instead of performing Yfiler analysis in all 6600 samples, which is time, labour, and resource intensive, the special force team in collaboration with the NFI applied a more effective approach. After the NFI had done Y-STR analysis in samples from the first set of 81 volunteers, allocated in the first collection box, already two Y-STR haplotype matches with the semen trace were obtained. Although, subsequent autosomal STR profiling excluded both men as likely suspects, this was a breakthrough finding. The Y-STR profile from the semen trace was so rare that it had not been ever recorded in any reference databases worldwide (including YHRD and a large unpublished Dutch Y-STR reference database); however, it showed up twice among the first 81 regional men analysed. By luck and thanks to the use of Y-STR profiling, the team had traced the paternal family of the unknown perpetrator after having analysed the first 81 regional men only. This result confirmed the previous assumption that led to the regional DNA dragnet, that the unknown perpetrator likely was a local man; at least his close and/or distant paternal relatives were, indeed, living in this area.

**Table 1:** Y-STR markers widely used in forensic DNA analysis

Y-STR marker	Commercial Y-STR kits/Non-commercial Y-STR sets					
	Minimal haplotype^c^	PowerPlex^®^ Y^a^	AmpF*l*STR^®^ Yfiler^®b^	PowerPlex^®^ Y23^a^	Yfiler^®^ Plus^b^	RM Y-STR set^c^
DYS19	✓	✓	✓	✓	✓	
DYS385a/b	✓	✓	✓	✓	✓	
DYS389I	✓	✓	✓	✓	✓	
DYS389II	✓	✓	✓	✓	✓	
DYS390	✓	✓	✓	✓	✓	
DYS391	✓	✓	✓	✓	✓	
DYS392	✓	✓	✓	✓	✓	
DYS393	✓	✓	✓	✓	✓	
DYS437		✓	✓	✓	✓	
DYS438		✓	✓	✓	✓	
DYS439		✓	✓	✓	✓	
DYS448			✓	✓	✓	
DYS456			✓	✓	✓	
DYS458			✓	✓	✓	
DYS635			✓	✓	✓	
Y-GATA-H4			✓	✓	✓	
DYS481				✓	✓	
DYS533				✓	✓	
DYS549				✓		
DYS570				✓	✓	✓
DYS576				✓	✓	✓
DYS643				✓		
DYS449					✓	✓
DYS460					✓	
DYS518					✓	✓
DYS627					✓	✓
DYF387S1a/b					✓	✓
DYS526a/b						✓
DYS547						✓
DYS612						✓
DYS626						✓
DYF399S1						✓
DYF403S1a/b						✓
DYF404S1						✓

^a^Promega

^b^Thermo Fisher Scientific

^c^Non-commercial

Moreover, instead of continuing with Y-STR profiling systematically in a box-by-box manner until all 6600 volunteers were analysed, the special force team then performed genealogy research in public registry archives on the two Y-STR matching volunteers. What they found was that these two men, who had different surnames, shared the same paternal ancestor at a time before the Dutch were forced to have their surnames registered during the Napoleon occupation. This explains why they share the same Y-STR haplotype but carry different surnames. The team then used this knowledge for effectively prioritizing the subsequent Y-STR analysis. They selected samples from volunteers with these two surnames, which could indicate that they belong to the perpetrator’s extended paternal family. By applying this approach, Y-STR profiles were never generated on thousands of collected samples, which saved time, money, and resources. Moreover, this intelligence-driven approach secured the privacy of thousands of volunteers, whose collected DNA samples were never analysed.

As may be expected for a rural area such as Friesland where typically male relatives stay in the region, the team identified several volunteers who matched the Y-STR haplotype from the semen trace. Aiming to further guide the investigation to the perpetrator’s close (instead of distant) relatives, it was decided that additional Y-STR markers need to be analysed in the DNA samples from the semen trace as well as from all volunteers with matching Yfiler Y-STR profiles. The special force team ordered the analysis of additional 38 Y-STR markers to be performed by Ronny Decorte from the Department of Forensic Biomedical Sciences of the KU Leuven. Moreover, the NFI performed profiling of 13 Y-STRs known to have an untypically high mutation rate, so-called rapidly mutating (RM) Y-STRs. The scientific and investigative motivation behind this decision was based on the expectation that by increasing the number of Y-STRs, particularly using RM Y-STRs, the chance to detect Y-STR mutations that allow separating distant male relatives from close ones increases, which, in turn, decreases the suspect pool. Distant relatives identified because of observed mutations leading to non-matching extended Y-STR profiles could thus be excluded from being relevant to the case, whereas close relatives with matching extended Y-STR profiles provided focused leads in the search for the unknown perpetrator.

However, to the surprise of everybody in the team, it turned out that one of the volunteers with a Y-STR profile match also showed an autosomal STR profile match with the semen trace. This finding provided strong evidence that this particular man was the donor of the semen trace. Soon after his subsequent arrest, Jasper S. of Dutch European ancestry and from Oudwoude located 2.5 km away from the murder site confessed that he had raped and murdered Marianna Vaatstra during the night of April 30, 1999. As a result, he was found guilty by the court in Leeuwarden and sentenced for 18 years in prison on April 2013, 14 years after the murder. Because the power of the applied Y-STR dragnet for familial searching has widely been communicated, he likely expected that several of his close and/or extended family members from the region would participate in the DNA dragnet, and would, therefore, reveal his identity eventually. He may have thought that direct participation was his only chance to escape, by hoping that the authorities, having to collect thousands of DNA profiles for the first time in Dutch history, would eventually make mistakes. This could explain why he did not show-up for voluntary sampling at his designated place during the first days of sampling, but only participated in the last days of voluntary sample collection at a different collection place. In the end, Jasper S. was identified as the murderer of Marianne Vaatstra, because he directly participated in the DNA dragnet. Obviously, under the scenario of direct participation, his DNA identification would also have occurred when the conventional autosomal STRs had been used instead of Y-STRs. However, it remains unclear whether he would, indeed, have voluntarily participated in an autosomal STR dragnet; the increased power of relative identification with the Y-STR dragnet had widely been communicated. In any case, it can be expected that the combined approach of a dense Y-STR dragnet, genealogy investigation, and additional Y-STR testing, particularly the use of RM Y-STRs, would have allowed the special force team to trace him eventually, even if he had not participated in the Y-STR dragnet.

This case particularly demonstrates the necessity and suitability of forensic Y-chromosome DNA analysis, which is discussed in more detail in the following chapters. One aspect not being further outlined below is the routine forensic use of Y-chromosome DNA for inferring the biological sex of a trace donor. In brief, biological sex information can be inferred from DNA via analysing genetic loci located on both sex chromosomes. In addition to STRs, all currently used commercial autosomal STR profiling kits target a small fragment of the amelogenin gene, which has a length polymorphism between its X-chromosome and Y-chromosome copies that is detected in the analysis. However, the use of amelogenin as sole sex marker in forensic DNA testing has repeatedly been criticized (Brinkmann 2002; Santos et al. 1998; Steinlechner et al. 2002; Thangaraj et al. 2002) due to rarely occurring Y-chromosome deletions that include the amelogenin locus, which makes such males to appear as female instead in the test outcomes. Nevertheless, until now, amelogenin remains the only sex marker in current commercial DNA profiling kits, but considerations should be given to including more sex-indicating DNA markers in the future.

## Y-STRs for paternal lineage identification

Standard DNA profiling using sets of well-selected, largely standardized, highly polymorphic autosomal STRs, is very suitable for identifying a donor of a single-source crime scene trace, as long as this person’s STR profile is already known to the investigating authorities. Nowadays, such knowledge typically comes from forensic DNA databases, where STR profiles of convicted crime offenders are stored and STR profiles obtained from crime scene traces are compared with to look for a match. Obviously, this comparative autosomal STR profile matching for human identification is not successful for completely unknown perpetrators, whose STR profiles are not yet available. Moreover, autosomal STR profiling is compromised in cases where more than one person has contributed to a crime scene trace (multiple-source samples). Only under certain favourable circumstances, such as one donor contributing much more DNA to the mixed stain than the other(s), it is possible to single out complete autosomal STR profiles from such mixed stains, while in many such cases, it is not. There is one type of crime cases, where multiple-source material typically comes from male and female contributors, and the to-be-identified male usually is the minor contributor. This is cases of sexual assault, where DNA analysis needs to be performed on vaginal swabs to identify the male rapist. In such cases, the autosomal STR profile of the female major contributor from her excess of epithelial cells is known to the investigators from the victim’s reference sample. Nevertheless, due to preferential PCR amplification of the major DNA component, and due to potential allele sharing between victim and perpetrator, it is often difficult, and in many cases impossible, to single out the autosomal STR profile of the male perpetrator from such mixed material. This is where Y-chromosome STR profiling comes into play, as only the male perpetrator, but not the female victim, carries a Y-chromosome.

Starting in 1992 with the publication of the first polymorphic STR discovered on the non-recombining part of the Y-chromosome (Roewer et al. 1992), and its immediate application to forensic casework (Roewer and Epplen 1992), more and more Y-STR markers were subsequently developed for forensic Y-STR haplotyping (Gopinath et al. 2016; Hall and Ballantyne 2003; Hanson and Ballantyne 2004, 2007; Kayser et al. 1997; Krenke et al. 2005; Lim et al. 2007; Mulero et al. 2006; Rodig et al. 2008; Thompson et al. 2013; Vermeulen et al. 2009). Up to 27 markers are currently included in commercial Y-STR kits [Yfiler Plus, Thermo Fisher Scientific (Gopinath et al. 2016)] (Table 1). Due to the achieved high haplotype diversity, these tools allow for the characterization of a paternal lineage with high, albeit not maximal, degree of certainty, especially when the tested sample donor comes from an outbred population (Purps et al. 2014; Vermeulen et al. 2009). Moreover, these commercial Y-STR kits allow the detection and characterization of DNA from males in mixed stains with high excess of DNA from females, also in cases with very low quantities of DNA from the minor male contributor as typical in material from sexual assault (Purps et al. 2015). Recommendations on forensic analysis of Y-STRs have been established by the DNA Commission of the International Society of Forensic Genetics (Gill et al. 2001; Gusmao et al. 2006), and the Y-STR kits have forensically been validated (Gopinath et al. 2016; Krenke et al. 2005; Mulero et al. 2006; Thompson et al. 2013). This allows forensic practitioners not only to exclude male suspects from being involved in a crime via non-matching Y-STR haplotypes, but also to identify the paternal lineage that a trace donor belongs to via matching Y-STR haplotypes (Roewer 2009). For example, a recent study of hundreds of sexual assault cases, where Y-STR haplotyping had been applied together with standard autosomal STR profiling, showed that one tenth of these cases would have remained inconclusive without the use of Y-STRs, and furthermore, Y-STR haplotyping was three times more suitable to identify multiple male contributors than autosomal STR profiling (Purps et al. 2015).

Because of the completely linked inheritance of loci on the non-recombining part of the Y chromosome, the product rule of multiplying single locus allele frequencies cannot be applied, and, therefore, complete haplotype frequencies are needed for estimating Y-STR-based match probabilities. As Y-STR haplotypes are by magnitudes more variable than single autosomal STR loci, Y-STR haplotype reference databases must be by magnitudes larger than autosomal STR allele reference databases to provide reliable frequency estimates. The largest and most widely used Y-STR haplotype reference database is the YHRD (Willuweit and Roewer 2015), which currently (January 2017, Release 52) includes between 178,171 and 16,577 Y-STR haplotypes depending on the marker set. Searching a Y-STR haplotype obtained from a crime scene trace against the reference database provides the frequency of the haplotype needed for calculating the match probability.

As expected, a paternal lineage can be more accurately characterized via Y-STR haplotyping that the more Y-STR markers are considered. However, once a certain Y-STR set has been identified, as was the first set of nine markers referred to as Minimal Haplotype (Table 1) (Kayser et al. 1997), adding additional Y-STRs not necessarily improves paternal lineage resolution. Classically, population genetic studies are carried out to identify Y-STR markers suitable for paternal lineage identification based on diversity measures (Hanson and Ballantyne 2004, 2007; Kayser et al. 1997; Vermeulen et al. 2009). However, the general disadvantage of such diversity-driven approach is that the obtained Y-STR set is highly suitable for paternal lineage differentiation on some populations (i.e., the ones tested or those similar to the ones tested), but may not be in others (i.e., those with distant ancestry to the ones tested). For example, South African populations showed low levels of haplotype diversity with the 9 Y-STRs from the Minimal Haplotype (Leat et al. 2004), which could be improved drastically with 11 Y-STRs (all except one marker being different than MH) selected from a South African population diversity data set of 45 Y-STRs (D’Amato et al. 2011).

However, some Y-STR markers seem more suitable than others to increase haplotype diversity and lineage resolution across populations. For instance, genotyping the 590 unrelated males from 51 worldwide populations included in the CEPH-HGDP panel for 67 Y-STRs, including the 17 standard Y-STRs from two commercial sets (PowerPlex-Y and Yfiler, see Table 1) and 49 non-standard Y-STRs (Lim et al. 2007), demonstrated that paternal lineage differentiation was increased over the commercial sets, globally and in all continental regions (except North Africa but represented by only a single population sample of small sample size) (Vermeulen et al. 2009). Six of the non-standard Y-STRs stood out in their value for lineages differentiation, in general, and for improving lineage differentiation when combined with standard Y-STRs (Vermeulen et al. 2009). Not surprisingly, these 6 Y-STRs were chosen by Promega (together with other markers already included in the Yfiler kit by Thermo Fisher Scientific) to expand and improve their PowerPlex-Y kit (Table 1), resulting in today’s PowerPlex-Y23 kit (Thompson et al. 2013) (Table 1).

The general disadvantage of this diversity-driven approach for selecting useful Y-STRs can be overcome in part when using mutation rate estimates to select suitable markers. Although these two approaches are not entirely independent of one another, the mutation-driven approach only considers the actual genetic changes produced by mutations, while the diversity-driven approach additionally considers other factors such as migration, fluctuating population size, genetic drift, putative selection, etc., which can complicate the identification of suitable markers. However, the success of both approaches largely depends on study sample size. Even though all male-specific Y-STR loci are genetically linked, given the underlying mutational process of strand slippage during DNA replication, different Y-STR loci mutate independently from each other. The mutation rate of Y-STRs is mostly determined by the number of repeats, particularly the number of repeats in non-interrupted repetitive stretches, where more repeats lead to more DNA slippage during replication (Ballantyne et al. 2010; Kayser et al. 2004). Y-STRs with a higher mutation rate are expected to be generally more suitable for differentiating paternal lineage compared to those with a low mutation rate. For instance, this was seen in the aforementioned study (Vermeulen et al. 2009), where samples from various deep-rooted pedigrees were also analysed to get a preliminary indication of the mutation rates of the 49 non-standard Y-STRs used. Notably, two of them (DYS570 and DYS576) stood out with much higher mutation rates compared to all other tested Y-STRs, including those from the commercial kits. These two were among the best 5 of all 67 tested Y-STRs, and among the best 6 of all 49 non-standard Y-STRs, eventually chosen for the PowerPlex-Y23 kit.

A later comprehensive mutation rate study of 186 Y-STRs in nearly 2000 DNA-confirmed father–son pairs not only confirmed the high mutation rate of these two Y-STRs, but identified 11 additional Y-STRs with similarly high mutation rates (i.e., a few mutations per 100 generations per each locus) (Ballantyne et al. 2010). These 13 RM Y-STRs (Table 1) are extremely useful for paternal lineage differentiation and identification (Ballantyne et al. 2012). For instance, in a large multicenter study including 12,272 unrelated males from 111 global populations, 12,156 (99%) were differentiated by unique RM Y-STR haplotypes (Ballantyne et al. 2014). For comparison, 6975 (89.6%) of a subset of 7784 unrelated men from 65 global population were separated with the Yfiler kit, while 7714 (99.1%) were separated with the RM Y-STR set (Ballantyne et al. 2014). The value of RM Y-STRs to differentiate between close and distant male relatives will be discussed below.

Mutation rates of the same Y-STR loci can differ between populations; however, strong and thus practically relevant differences could only develop in populations that experienced an extreme bottleneck and founder effect followed by strong isolation. This is rare, perhaps, with the exception of remote island groups. Moreover, strong mutation rate differences would only occur if the founding males either predominantly carried Y-STR alleles with particularly long or with particularly short stretches of uninterrupted repeats favouring or disfavouring Y-STR mutations, respectively (Ballantyne et al. 2010). Such founder selection based on extremes in Y-STR repeat length is very unlikely to occur by chance or any other means. Although Y-STR mutation rate differences between and/or within populations have been observed for the same loci (Goedbloed et al. 2009), they are rather small and can likely be explained by stochastic effects due to the rarity of occurring mutations given the small sample sizes used in some studies.

Overall, Y-STR haplotyping is very useful both for excluding suspects from involvement in a crime by demonstrating non-matching haplotypes, and for identifying groups of male relatives belonging to the same paternal lineage by demonstrating haplotype matches. However, commercial Y-STR kits are not suitable for male individual identification, because male relatives typically share the same resulting haplotype. Consequently, a match probability estimated for a Y-STR haplotype established with any of these kits not only applies to the tested suspect, but similarly to all of his untested male paternal relatives. It will then be up to the police to find out if, indeed, the matching suspect, or instead any of his close or distant male relatives, has left the trace at the crime scene. What is a disadvantage of Y-STRs for individual identification purposes serves as an advantage for paternity testing, other types of kinship testing, and for familial searching (as applied in the Vaatstra case).

## Y-STRs for paternity testing, kinship analysis, and familial searching

Because Y-STR haplotypes are shared between paternally related men belonging to the same paternal lineage, Y-STR haplotyping is suitable for solving paternity disputes of male offspring, other types of paternal kinship questions, and for familial searching. It is also suitable to male identification cases involving human remains such as in disaster victim and missing person identification where only distant relatives are available. In paternity testing, Y-STR haplotyping is particularly suitable in deficiency cases, where the putative father of a male child is deceased and not available for DNA testing. With autosomal DNA profiling, the paternity of the unavailable putative father to the child can be established or rejected with the necessary high degree of certainty only if both parents of the deceased putative father are available for testing. If only one or none of the paternal grant parents of the male child are available, Y-STR profiling comes into play as long as any male relative of the deceased putative father is available for analysis. By use of standard Y-STRs with low–medium mutation rates [i.e., one or a few mutations every 1000 generations per each locus, (Goedbloed et al. 2009)], male relatives of the deceased putative father will share the same Y-STR haplotype with the putative father, and thus with his son, in case of biological paternity. Obviously, RM Y-STRs characterised by increased mutation rates are not suitable for paternity and kinship testing, as the mutations observed with increased probabilities will trouble the estimation of paternity/kinship probabilities.

As long as enough Y-STRs with low–medium mutation rates are analysed, allowing the clear characterization of the paternal lineage to which the putative father’s paternal relative and the son belong, finding the same haplotype indicates biological paternity. The strength of probability of paternity will depend on the frequency of the Y-STR haplotype observed. The same applies in kinship analysis where the paternal relationship of one or more males is to be established or tested from hypotheses based on family record or archive information. However, even with such low–medium mutation rates, the chance of observing haplotypes that are different at certain Y-STRs due to rare mutations will generally increase the more Y-STRs are used. On the other hand, more Y-STRs can typically characterise and identify a paternal lineage better (see above), resulting in a dilemma in cases where haplotype differences are observed to decide between paternity/kinship with mutations versus non-paternity/non-kinship. For instance, in a Yfiler study using 1730 father-son pairs and finding a total of 84 mutations, one pair was found with mutations at 3 of the 17 Y-STRs, while two pairs with mutations at two Y-STRs, respectively (Goedbloed et al. 2009). Moreover, as it may be expected, when these father-son pairs were analysed for additional 169 Y-STRs, both the number of pairs with mutations at multiple Y-STRs, and the number of Y-STRs at which mutations were observed, increased (Ballantyne et al. 2010). In this extended study, 123 father–son pairs were found with mutations at 3 Y-STRs, 42 pairs with mutations at 4 Y-STRs, and 3 pairs with mutations at 5, 6, 7, and 8 Y-STRs (Ballantyne et al. 2010). Therefore, instead of applying a fixed rule for excluding from paternity (or other kinship questions) based on exclusion constellations of the minimum of three Y-STRs, as argued previously (Kayser and Sajantila 2001), it is more sensible to use a flexible model. Such model shall consider the total number of Y-STRs analysed, their locus-specific mutation rate estimates, and the repeat number differences of the non-matching alleles observed. The latter is indicated, because the majority of Y-STR mutations represent single repeat changes (Ballantyne et al. 2010).

As long as the person in question is a male, the non-recombining nature of male-specific Y-chromosome markers principally also allows to solve historical cases of paternity, or other types of paternal kinship dispute, as well as identification cases many generations after they occurred, which is impossible with recombining autosomal DNA. In historical identification cases, DNA from the remains of the historical man as well as from his living paternal relative assumed from family records must be available for Y-chromosome DNA analysis. In historical paternity cases, either DNA from the remains of the putative father and the son, or from living male descendent from both, as assumed from family records, must have available for Y-chromosome DNA analysis. When matching Y haplotypes are observed, true biological paternity/kinship or identification can be assumed, while different haplotypes indicate non-paternity or kinship. However, when observed haplotype differences are too many and/or too large in repeat number differences to be explainable by mutations, given the mutation rates of the Y-STRs and the number of separating meiosis in the family line, it is typically difficult, if not impossible, to find out at which male in the family line the non-biological paternity occurred. Moreover, matching haplotypes do not necessarily permit the conclusion of paternity or identity of the historical men, because his close relatives living at the time would likely have shared the same Y-chromosome haplotype, and, therefore, could have been the father/wanted men with the same probability estimated from the Y haplotype.

Two examples of historical paternity and identification cases where Y-chromosome DNA was applied are mentioned here for illustration. In the paternity dispute of former US President Thomas Jefferson (1743–1826), Y-STR and Y-SNP analysis demonstrated that several currently living male relatives of Thomas Jefferson share the same Y haplotype as a living descendent of Eston Hemings Jefferson, son of Sally Hemings—the President’s African American female slave (except for one repeat difference at one Y-STR, which could be easily explained by a mutation) (Foster et al. 1998). This indicates that President Jefferson had sired Eston Hemings Jefferson, or alternatively, his brother Randolph did; two scenarios such Y-chromosome analysis cannot differentiate. However, living male descendent of Thomas Corbin Woodson, the previously assumed full brother of Eston Hemings Jefferson, showed a very different Y haplotype, indicating that his biological father was a different man (Foster et al. 1998). In the identification case of King Richard III of England (1452–1485), various types of evidence including a complete match of the entire mitochondrial genome between the skeleton and an assumed living maternal relative of King Richard III gave a large likelihood that the skeleton is that of the King (King et al. 2014). However, the skeleton’s Y haplotype did not match that of the King’s living paternal relatives. Because of the overwhelming evidence in favour of identification, the Y discrepancy was concluded as indication of a false-paternity in any men of the extended family between the tested paternal relatives and the King (King et al. 2014).

The same principle of haplotype sharing between close and distant paternal male relatives as applied in paternity and kinship testing makes Y-STR haplotyping also suitable for familial searching, in forensic cases without autosomal DNA profile match (as applied in the Vaatstra case). However, different to paternity and other types of paternal kinship analysis, where only Y-STRs with low–medium mutation rates are suitable, Y-STR applications to familial searching may additionally require the use of RM Y-STRs (see also next chapter). This is in cases where haplotype matches based on low–medium mutating Y-STRs are seen with several persons (as in the Vaatstra case). Subsequently, RM Y-STRs need to be analysed, to separate-out the more distantly related male relatives identified by mutations, allowing to focus on the closely related ones highlighted by not showing mutations to guide the search for the unknown male perpetrator whose DNA was not available.

## Y-STRs for male relative differentiation towards male individual identification

Due to the low–medium mutation rates of most of their Y-STRs, the commercial Y-STR kits have limitations in differentiating paternal lineage in inbred population, where the proportion of distantly related males is increased. Moreover, they typically fail to separate male relatives belonging to the same paternal lineage, thus not allowing individual identification, as is strongly desired in forensic DNA analysis in general. A way out of this dilemma was indicated by the first discovery of RM Y-STRs with untypically high mutation rates (Ballantyne et al. 2010). In principle, it can be expected that with sufficient numbers of RM Y-STR markers available, close, and especially distantly related men will be separated by means of observed mutations. Thus, individual identification can be achieved while maintaining the advantages of Y-chromosome DNA analysis for male–female mixed stain resolution.

Empirical evidence of male relative differentiation with the full 13 RM Y-STR set has steadily increased over the past few years. The discrimination rates currently most-supported by available data are 27% for father–sons based on 2378 pairs (Ballantyne et al. 2014), 44% for brothers and grandfather–grandsons separated by 2 meioses based on 480 pairs (Adnan et al. 2016), 55% for cousins separated by three meiosis based on 308 pairs (Adnan et al. 2016), and 61% for male relatives separated by 4 meioses based on 277 pairs (Adnan et al. 2016). The most recent commercial Y-STR kits include two (PowerPlex-Y 23) and 6 (YfilerPlus) RM Y-STRs (Table 1). These kits, therefore, do not provide the full power of male relative differentiation as available with the complete set of 13 RM Y-STRs. Thus far, no commercial kit exists for all 13 RM Y-STRs, but non-commercial multiplex genotyping protocols are available (Alghafri et al. 2015; Ballantyne et al. 2012).

It is envisioned that if in a criminal case, a Y haplotype match is established with any of the commercial Y-STR kits, the full set of 13 RM Y-STRs shall be analysed to test whether the matching suspect, or his close or distant paternal male relatives, has left the trace at the crime scene. Furthermore, in constellations where there is a match of Y-STR haplotypes from commercial kits, and there is evidence that a close relative of the known suspect (such as a brother) may rather be the trace donor, reference DNA samples of both males shall be tested for the complete set of 13 RM Y-STRs. In case that a separating mutation at any of the RM Y-STRs is observed in any of the two reference DNA samples, the crime scene trace shall be analysed for RM Y-STRs, to establish to whom of the two men the evidence RM Y-STR haplotype matches. Although the current set of 13 RM Y-STRs has limitations in differentiating relatives, especially close ones (see numbers of male relative discrimination mentioned above), more RM Y-STRs may be identified in the future that will allow further increasing male relative differentiation rates, and may even eventually achieve individual identification of a man from Y-chromosome DNA analysis.

The ultra-high rate at which unrelated males can be differentiated with RM Y-STRs leads to the practical problem of putting statistical weight on a RM Y-STR haplotype match. Clearly, the more polymorphic a Y-STR haplotype is, the larger the haplotype reference database needs to be to deliver reliable frequency estimates. The problem of singletons in Y-STR reference databases has already been noticed for haplotypes established from commercial Y-STR kits. Such Y-STR haplotypes, however, are much less polymorphic than those generated from the full set of 13 RM Y-STRs (Ballantyne et al. 2014), but the size of the YHRD is already large with currently (as of January 2017) 139,104 PowerPlex-Y haplotypes and 126,409 Yfiler haplotypes included (https://yhrd.org, Release 52). Despite this enormous size that could only be achieved via collaboration of the global forensic DNA community over decades, many Y-STR haplotypes obtained in routine forensic practise are not yet included in the YHRD. This poses a statistical problem on how to get reliable haplotype frequency estimates needed for calculating match probabilities. Forensic statisticians have been trying to develop solutions (Andersen et al. 2013; Brenner 2010; Buckleton et al. 2011), but no consensus on the most suitable method has been reached thus far. Clearly, this problem becomes more severe for haplotypes based on RM Y-STRs that are much more variable than those obtained from commercial Y-STR kits (Ballantyne et al. 2014), including the most recent kits (Purps et al. 2014).

## The Y chromosome for inferring paternal bio-geographic ancestry

As mentioned above in the context of the Vaatstra case, the Y chromosome is highly suitable to provide information about the geographic region a person’s paternal ancestors originate from, i.e., bio-geographic ancestry. Forensic DNA testing for bio-geographic ancestry is useful in cases where autosomal STR profile matches are lacking, because the perpetrator is completely unknown to the investigators. In such cases, bio-geographic ancestry information obtained from evidence DNA [at best in combination with information regarding externally visible characteristics and age (Kayser 2015)] can guide police investigations towards finding unknown perpetrators (Phillips 2015). Similarly, DNA testing on bio-geographic ancestry can be useful in missing person cases, including disaster victim identification cases, without any knowledge about the possible identity of the person to whom the biological remains belong.

In general, the suitability of Y-chromosome DNA for inferring paternal bio-geographic ancestry comes from its escape from recombination, as it is also seen for maternal ancestry with maternally inherited mitochondrial (mt) DNA. Under the absence of recombination, once a mutation has occurred, it is not removed from the gene pool, unless no male (or male and female in case of mtDNA) offspring exists. Both uniparentally inherited parts of the human genome (Y and mt) are, therefore, more prone to genetic drift, which can produce genetic differences between geographic regions simply by chance. Further contributing to the suitability of the Y-chromosome for ancestry inference is certain elements of human culture, such as patrilocal residence and polygyny, which increase Y-chromosome differences over geographic distance. For decades, Y-chromosomal DNA polymorphisms were explored to trace bio-geographic ancestry of individuals and populations, in the beginning mostly from an evolutionary perspective to understand population origins and migration history worldwide (Underhill and Kivisild 2007). Such research produced a wealth of knowledge on the geographic distribution of Y-chromosome genetic diversity, which serves as the basis for the forensic applications of paternal bio-geographic ancestry inference, particularly for Y-SNPs.

Because of their about 100,000 lower mutation rates relative to most Y-STRs (Ballantyne et al. 2010; Xue et al. 2009), geographic ancestry signatures are kept much longer at Y-SNPs before being diluted via mutations, relative to Y-STRs. Therefore, Y-SNPs are generally more suitable for paternal bio-geographic ancestry inference than Y-STRs. It is widely assumed that modern humans go back to a single recent common origin in Africa, that they first left Africa about 100,000 years, and arrive in the different continental regions between 60,000 and 15,000 years ago, depending on the region. This history equals enough generation steps to allow Y-chromosome mutations generating continental differences at various Y-SNPs. Furthermore, subsequent population movements, male-driven cultural traits, genetic drift, and various other factors have produced Y-SNP frequency differences between geographic regions and, albeit less pronounced, between subregions.

In recent years, more and more large-scale resequencing studies using massively parallel sequencing (MPS) technologies, also referred to as next-generation sequencing (NGS), have produced a large number of newly discovered Y-SNPs (Batini et al. 2015; Francalacci et al. 2013; Hallast et al. 2015; Scozzari et al. 2014; Trombetta et al. 2015), much larger than previously found with other technologies (Karafet et al. 2008). They are placed into their phylogenetic position via routinely updated global Y-chromosome trees made available as open resource by the International Society of Genetic Genealogy (ISOGG) (http://isogg.org/tree/index.html). A minimal reference phylogeny for the human Y-chromosome, representing an abbreviated version of the Y-tree showing only the principal branches together with the geographic regions of predominant occurrence, is available via Phylotree-Y (http://www.phylotree.org/Y/tree/index.htm) (van Oven et al. 2014). For orientation, Table 2 provides a selected list of Y-SNP haplogroups with their geographic regions of predominant occurrence that are informative for paternal bio-geographic ancestry inference. Moreover, various Y-SNP genotyping tools suitable to low-quantity and low-quality DNA have been developed for forensic and other applications (such as anthropology and genealogy) (Brion et al. 2005; Gomes et al. 2010; van Oven et al. 2011, 2012, 2013). Due to the SNaPshot technology used, these tools have restrictions in the number of Y-SNPs analysed simultaneously, providing limitation on the geographic resolution at which paternal bio-geographic ancestry can be obtained with such tools. Many Y-SNPs together with their respective genotyping tools are available that allow paternal bio-geographic ancestry inference on the level of continental resolution. For some continental regions, such as Europe, Y-SNPs also allow subregional inference of paternal ancestry (Balaresque et al. 2010; Batini et al. 2015; Cruciani et al. 2011). However, for many of the recently discovered and already phylogenetically mapped Y-SNPs, population data are lacking, so that their suitability for paternal bio-geographic ancestry testing needs to be established in the future via the generation of population data to reveal their geographic distributions.

**Table 2:** Selective list of Y-SNP-based haplogroups informative for paternal bio-geographic ancestry inference worldwide

Haplogroup	Defining bi-allelic marker(s)	Respective rs number(s)	Major geographic area(s) of occurrence
A00-L1086	L1086; L1159; L1284	N/A; N/A; N/A	Central Africa
A0-V148	V148; V166; L896; L991	rs181335666; rs187287389; N/A; N/A	Central Africa, West Africa
A1-M31	M31; P82; V4	rs369315948; N/A; rs187409543	West Africa, North Africa
A2-V50	V50; L602	rs189205028; rs576471146	Southern Africa, Central Africa
A3-M32	M32	rs558241924	East Africa, Southern Africa
B-M60	M60; M181; V244	rs2032623; rs2032599; rs112298449	Central Africa, Southern Africa, East Africa
D-M174	M174; CTS94; JST021355	rs2032602; rs199881488; rs2267802	East Asia
E-M96	M96; M40; P29	rs9306841; rs9786608; rs60115999	Africa, West Asia, Southern Europe
E-V13	V13; V36	rs368031074; rs371443469	Southern Europe
E-M293	M293	rs9341316	Southern Africa
C-M130	M130; M216	rs35284970; rs2032666	Central Asia, Northern Asia, North America, East Asia, Southeast Asia, Wallacea, Near Oceania, Remote Oceania, Australia
C-M8	M8; M105	rs3899; rs2032612	Japan
C-V20	V20	rs182352067	Southern Europe
C-M356	M356	N/A	South Asia, Central Asia
C-B65	B65	rs374541802	Indonesia, Philippines
C-M38	M38	rs369611932	Wallacea, Near Oceania, Remote Oceania
C-M208	M208	rs2032659	Near Oceania, Remote Oceania
C-P33	P33	N/A	Remote Oceania
C-PH41	PH41; PH338; PH4186; PH4682	N/A; N/A; N/A; N/A	Australia
C-M217	M217; P44; Z1453	rs2032668; N/A; N/A	Central Asia, Northeast Asia, Northern Americas
C-P39	P39	N/A	Northern Americas
G-M201	M201; P257	rs2032636; rs2740980	West Asia, Europe, Central Asia
H-L901	L901; M3035	rs567848586; rs74378870	South Asia
I-M170	M170; M258; U179	rs2032597; rs9341301; rs2319818	Europe, West Asia
J-M304	M304; P209	rs13447352; rs17315835	West Asia, North Africa, Horn of Africa, Southern Europe, Central Asia, South Asia
L-M20	M20	rs3911	South Asia, West Asia
M-P397	P397; P399; PR2099	N/A; N/A; rs369017623	Wallacea, Near Oceania, Remote Oceania, Australia
M-P34	P34	N/A	Near Oceania
M-M10072	M10072; FGC38729; Z33118	rs566812523; N/A; rs368850080	Australia
N-M231	M231	rs9341278	Northern Asia, Northern Europe
O-M175	M175; P186	rs2032678	East Asia, Southeast Asia, Remote Oceania
Q-M242	M242	rs8179021	Northern Asia, Central Asia, Americas
Q-M3	M3	rs3894	Americas
Q-Z780	Z780	N/A	Americas
R-M207	M207	rs2032658	Europe, West Asia, Central Asia, South Asia, North Africa, Central Africa
R-M458	M458	rs375323198	Eastern Europe, Caucasus region
R-Z284	Z284	N/A	Northwest Europe
R-Z93	Z93	rs566323605	South Asia, Central Asia
R-V88	V88	rs180946844	Africa
R-M412	M412	rs9786140	Western Europe
R-M479	M479	rs372157627	South Asia, Central Asia
S-M254	M254	rs9341297	Near Oceania
T-M184	M184	rs20320	West Asia, Horn of Africa, North Africa, Southern Europe, South Asia

Some Y-SNP-based haplogroups with strong frequency differences between geographic (sub)regions display a strong-enough correlation with their associated Y-STR haplotype diversity, so that the geographic regions indicated by the Y-SNP haplogroup can also be inferred from associated Y-STR haplotypes (as performed in the Vaatstra case). To cover more of the geographic information of a Y-STR haplotype, a nearest neighbour Y-STR haplotype search in the reference database can help, as this would take mutation steps into account. Well-known examples are the major haplogroups R1b indicating Western European paternal ancestry and R1a indicating Eastern European paternal ancestry. However, not many Y-STR haplotypes with strong geographic signatures are known. Notably, the YHRD has recently been expanded to Y-SNP data and currently (as of January 2017, Release 52) includes 20,187 Y-SNP profiles (https://yhrd.org).

## The future of forensic Y-chromosome DNA analysis

In recent years, commercial Y-STR kits have seen an improvement in the number of Y-STR markers included, enabling increased paternal lineage resolution. However, these kits cannot differentiate all unrelated men in a population, nor do they allow the discrimination of related men. It, therefore, is envisioned that future commercial Y-STR kits will include more markers, particularly more RM Y-STRs. However, including more RM Y-STRs will lead to a further increasing problem of putting a statistical weight on an observed haplotype match because of the ultra-high diversity of RM Y-STR haplotypes requiring extremely large reference databases for estimating a somewhat reliable frequency of the matching haplotype needed for estimating the match probability, unless new statistical solutions will be developed.

Moreover, the two different forensic applications of Y-STR haplotyping, on one hand paternal lineage identification, for which Y-STRs with high mutation rates are most suitable, and on the other hand paternal/kinship determination and familial searching, for which Y-STRs with low-medium mutation rates are most suitable, require that both types of Y-STR markers need to be considered in adequate numbers in future commercial kits. However, including many more than 27 Y-STRs, as is currently the maximum in commercial Y-STR kits, is approaching the limits of fluorescence-based fragment length analysis with capillary electrophoresis (CE), which represents the method of choice for routine forensic Y-STR analysis. This can be overcome with more fluorescence dyes being employed, requiring new chemistry and new instrumentation being developed. Alternatively, separate Y-STR kits for the two forensic applications could be developed, which would at least reduce the CE-based multiplexing problem by half. Targeted MPS technologies may serve as an alternative for multiplexing large numbers of Y-STRs, as already applied for autosomal STRs, Y-STRs, and SNPs with the ForenSeq kit (Illumina). However, the current MPS platforms suitable for forensic DNA analysis come with strong size limitations on the sequenced fragment length. Although the obtained short sequencing reads are suitable for many Y-STRs with low–medium mutation rates, they are not sufficient for RM Y-STRs with long repetitive stretches. Hence, MPS technologies enabling the sequencing of longer fragments from low-quality/quantity forensic DNA will have to be developed.

Regarding forensic ancestry testing using Y-chromosome markers, future work needs to provide more knowledge about the geographic distribution of many of the recently discovered Y-SNPs, to establish how useful they are for improving the geographic resolution of paternal ancestry inference. It is expected that such knowledge will allow paternal bio-geographic ancestry inference to be moved from the current level of mostly continental resolution to a much more detailed geographic resolution. As with Y-STRs, also for Y-SNPs, the limitation in multiplexing capacity of the genotyping technologies currently used in forensic DNA analysis has to be overcome, to take full advantage of the large number of Y-SNPs needed for inferring bio-geographic ancestry on a detailed level. Here, current targeted MPS technologies are highly promising because of their large multiplex capacity together with their short sequencing reads, given the single base pair nature of Y-SNPs. For example, a recent proof-of-principle study demonstrated that 530 Y-SNPs can be analysed simultaneously in a single targeted MPS run (Ralf et al. 2015). In any case, to achieve accurate bio-geographic ancestry inference of a person from its DNA, ancestry-informative SNPs from the Y chromosome for paternal ancestry inference need to be combined with those from mitochondrial (mt) DNA for maternal ancestry inference and from autosomal DNA for bi-parental ancestry inference. Such a combined genetic approach will allow inferring bio-geographic ancestry of admixed persons whose biological ancestors come from very different geographic regions, which is impossible with Y-chromosome DNA (or mt DNA) alone.

Finally, some authors, including in this special journal issue (Calafell and Larmuseau 2016), have suggested additional forensic applications of the human Y-chromosome than discussed above, for instance predicting a man’s surname from his Y-chromosome DNA (Calafell and Larmuseau 2016; King and Jobling 2009). In patrilineal societies, which most human societies are, surnames are transferred from the father to all his offspring, as is the human Y chromosome to his sons. In such societies, co-ancestry of surnames and Y-chromosomes is, therefore, expected. However, this comes under the prerequisites that a surname was not given to/taken by unrelated men, and that the rate of non-biological paternity is very small, at best zero, since the surname started to be used. In reality, many surnames have been given multiple times to unrelated men, so that men from different paternal families with different Y-chromosomes can have the same surname. The co-ancestry of surnames and Y-chromosomes is additionally decreased with any non-biological paternity occurring (around 1–2%), and through adoptions from outside the paternal family. So far, data evidence is limited and restricted to mainly three European countries, i.e., Great Britain (King et al. 2006), Ireland (McEvoy and Bradley 2006), and Spain (Martinez-Cadenas et al. 2016). Although strong co-ancestry has been observed for some rare surnames, common ones showed low or no Y-chromosome correlation (King et al. 2006; Martinez-Cadenas et al. 2016), which limits broad forensic applications. Nevertheless, the combined use of Y-chromosome data and surname information can be highly valuable in specific forensic cases, for instance when Y-chromosome-based familial searching is combined with genealogy investigation, as successfully applied for solving the murder case of Marianne Vaatstra.
